# The Salivary Microbiota of Patients With Primary Biliary Cholangitis Is Distinctive and Pathogenic

**DOI:** 10.3389/fimmu.2021.713647

**Published:** 2021-07-21

**Authors:** Longxian Lv, Huiyong Jiang, Xiaoxiao Chen, Qiangqiang Wang, Kaicen Wang, Jianzhong Ye, Yating Li, Daiqiong Fang, Yingfeng Lu, Liya Yang, Silan Gu, Jianing Chen, Hongyan Diao, Ren Yan, Lanjuan Li

**Affiliations:** State Key Laboratory for Diagnosis and Treatment of Infectious Diseases, Collaborative Innovation Center for Diagnosis and Treatment of Infectious Diseases, The First Affiliated Hospital, College of Medicine, Zhejiang University, Hangzhou, China

**Keywords:** liver, microbiota, autoimmune diseases, immunity, metabolites

## Abstract

The role of host-microbiota interactions in primary biliary cholangitis (PBC) has received increased attention. However, the impact of PBC on the oral microbiota and contribution of the oral microbiota to PBC are unclear. In this study, thirty-nine PBC patients without other diseases and 37 healthy controls (HCs) were enrolled and tested for liver functions and haematological variables. Saliva specimens were collected before and after brushing, microbiota was determined using 16S rDNA sequencing, metabolomics was profiled using Gas Chromatography-Mass Spectrometer (GC-MS), 80 cytokines were assayed using biochips, and inflammation inducibility was evaluated using OKF6 keratinocytes and THP-1 macrophages. Finally, the effect of ultrasonic scaling on PBC was estimated. Compared with HCs, PBC saliva had enriched taxa such as Bacteroidetes, *Campylobacter*, *Prevotella* and *Veillonella* and depleted taxa such as *Enterococcaceae, Granulicatella*, *Rothia* and *Streptococcus*. PBC saliva also had enriched sCD163, enriched metabolites such as 2-aminomalonic acid and 1-dodecanol, and depleted metabolites such as dodecanoic acid and propylene glycol. sCD163, 4-hydroxybenzeneacetic acid and 2-aminomalonic acid were significantly correlated with salivary cytokines, bacteria and metabolites. Salivary *Veillonellaceae* members, 2-aminomalonic acid, and sCD163 were positively correlated with liver function indicators such as serum alkaline phosphatase (ALP), aspartate aminotransferase (AST) and alanine aminotransferase (ALT). PBC salivary microbes induced more soluble interleukin (IL)-6 receptor α (sIL-6Rα), sIL-6Rβ and tumour necrosis factor ligand superfamily (TNFSF)13B from OKF6 keratinocytes, and PBC salivary supernatant induced more IL-6, IL-10, granulocyte-macrophage colony-stimulating factor (GM-CSF), chemokine (C-C motif) ligand (CCL)13, C-X-C motif chemokine (CXC)L1 and CXCL16 from THP-1 macrophages. Toothbrushing significantly reduced the expression of inflammatory cytokines such as IL-1β, IL-8 and TNF-α and harmful metabolites such as cadaverine and putrescine in PBC but not HC saliva after *P*‐value correction. The levels of ALP and bilirubin in PBC serum were decreased after ultrasonic scaling. Together, PBC patients show significant alterations in their salivary microbiota, likely representing one cause and treatment target of oral inflammation and worsening liver functions.

## Introduction

Primary biliary cholangitis (PBC) is a female-dominated autoimmune liver disease with an obscure aetiology (1). Recently, the human microbiota has been widely linked to PBC aetiopathogenesis (2–5). First, epidemiological studies identified urinary tract infection, predominantly by *Escherichia coli*, as a risk factor (2). Second, molecular mimicry supports microbes as PBC triggers. *E. coli* pyruvate dehydrogenase E2 components (PDC-E2) show significant homology to human PDC-E2 (6). *Novosphingobium aromaticivorans* has two lipoylated proteins with greater homology to human PDC-E2 (7). Mimicry peptides of human PDC‐E2 and evidence of cross-reactive antibodies were also observed for *Mycoplasma pneumonia*, *Lactobacillus delbrueckii* and *Mycobacterium gordonae* (2, 8). Third, microbial by-products may affect PBC. For example, Toll-like receptor ligands and CpG motifs can contribute to autoimmunity by stimulating the innate immune system (9). Interestingly, the gut microbiota of PBC patients was depleted of certain potentially beneficial bacteria, such as *Bacteroides eggerthii*, but was enriched for certain bacterial taxa containing opportunistic pathogens, such as *Klebsiella* (3, 4).

The oral microbiota is important for health and diseases (10). More than 700 microbial species colonize the buccal mucosa, tongue dorsum, dental plaque and other places, forming the largest microbiota outside the gut (11). These species have important physiological functions, including serving as biological barriers and stimulating and maintaining of host immunity. Additionally, the microbiota can pass through the oral cavity and affect *in vivo* organs, such as the gut and lung, because the teeth are effectively transmucosal organs (12). Microbes in saliva are the closest to those in the oral mucosa and can better reflect the composition and functions of oral microbiota. Almost a litre of saliva containing billions of microbes enters the digestive tract daily and contributes substantially to host immunity and metabolism (13). Additionally, saliva sampling is convenient and noninvasive, making saliva an important sample for research and diagnosis.

This study aimed to disclose alterations of the PBC salivary microbiota, identify the relationship of these alterations with oral immunity and metabolism and explore the potential mechanism of how an altered oral microbiota affects PBC.

## Materials and Methods

### Study Participants, Sample Collection and Handling

All PBC patients were diagnosed according to established criteria (14) and had no other serious diseases or health problems, such as obesity, diabetes, tumours, gastrointestinal disorders, hepatitis infection, fatty liver disease, alcoholic/drug-induced liver disease, cardiorenal dysfunction, circulatory system diseases, or nervous system diseases. Furthermore, the patients did not take antibiotics or probiotic drugs within the past month. The ethics committee of Zhejiang University approved this study. All the participants signed informed consent forms. First, samples were collected when the participants had not eaten breakfast, drunk water, brushed their teeth or used any oral cleanser. Saliva was drooled into a 50-mL sterile tube according to the Human Microbiome Project core microbiome sampling protocol A until at least 5 mL of saliva was collected. Second, all the participants cleaned their oral cavity, teeth and tongue according to the Bass method. After one hour, they rinsed their mouth five times and drank 100 mL to clean their throat again. After five minutes, saliva was collected again. Blood was drawn and separated into serum and blood cells. All the samples were stored at −80°C until use.

### Tests of Liver Function, Haematological Variables, and Mayo Risk Score

Liver function tests such as total protein (TP), albumin (ALB), globulin (GLOB), alkaline phosphatase (ALP), total bilirubin (TB), direct bilirubin (DB), indirect bilirubin (IB), and glutamyl transpeptidase (GGT) were assessed using a HITACHI 7600-210 automatic analyser (HITACHI, Tokyo, Japan). Haematological variables such as erythrocytes, haemoglobin, the platelet count, and the percentage and count of neutrophils, lymphocytes, monocytes, eosinophils and basophils were evaluated using a Sysmex XN-2000 system (Sysmex Corporation, Kobe, Japan). The Mayo risk score was calculated using the following equation: = 0.871 × log_e_ (serum bilirubin in mg/dL) − 2.53 × log_e_ (albumin in g/dL) + 0.039 × (age in years) + 2.38 × log_e_ (prothrombin time in seconds) + 0.859 oedema (0 = no oedema, no diuretic therapy; 0.5 = oedema, no diuretic therapy or no oedema, diuretic therapy; 1 = oedema and diuretic therapy) (15).

### Assay of Cytokines and Chemokines

The levels of 80 cytokines, namely, chemokine (C-C motif) ligand 1 (CCL1), CCL2, CCL3, CCL7, CCL8, CCL11, CCL13, CCL15, CCL17, CCL19, CCL20, CCL21, CCL22, CCL23, CCL24, CCL25, CCL26, CCL27, chemokine (C-X3-C motif) ligand 1 (CX3CL1), C-X-C motif chemokine 1 (CXCL1), CXCL2, CXCL5, CXCL6, CXCL8, CXCL9, CXCL10, CXCL11, CXCL12, CXCL13, CXCL16, granulocyte-macrophage colony-stimulating factor (GM-CSF), interferon (IFN)-γ, IFN-α2, IFN-β, interleukin (IL)-1β, IL-2, IL-4, IL-6, IL-10, IL-11, IL-12p40, IL-12p70, IL-16, IL-17A, IL-17F, IL-19, IL-20, IL-21, IL-22, IL-23, IL-25, IL-26, IL-27, IL-28A, IL-29, IL-31, IL-32, IL-33, IL-34, IL-35, macrophage migration inhibitory factor (MIF), matrix metalloproteinase (MMP)-1, MMP-2, MMP-3, osteocalcin, osteopontin, pentraxin-3, soluble cluster of differentiation 163 (sCD163), sCD40L, soluble IL-6 receptor α (sIL-6Rα), sIL-6Rβ, soluble tumour necrosis factor receptor (sTNFR)1, sTNFR2, tumour necrosis factor ligand superfamily member (TNFSF)8, TNFSF12, TNFSF13, TNFSF13B, TNFSF14, tumour necrosis factor α (TNF-α) and thymic stromal lymphopoietin, were measured using commercially available enzyme-linked immunosorbent assay kits (Bio-Rad Laboratories, Hercules, CA, USA) and the Bio-plex 200 System.

### DNA Extraction, PCR Amplification, 16S rDNA Sequencing and Data Processing

Five hundred microlitres of saliva was centrifuged at 3,000 rpm for 10 min. Pellets were used to manually extract genomic DNA as described previously (16). We amplified the V3-V4 region of the 16S rRNA gene using the primers 338F (5’-ACTCCTACGGGAGGCAGCAG-3’) and 806R (5’-GGACTACHVGGGTWTCTAAT-3’). PCR amplifications were conducted under the following cycling conditions: 98°C for 30 s; 35 cycles of 98°C for 10 s, 52°C for 30 s and 72°C for 45 s; and a postcycling extension at 72°C for 10 min. From the DNA extraction to the PCR process, ultrapure water, instead of a sample solution, was used as a negative control to exclude the possibility of false-positive results. The PCR products were purified using AMPure XT beads (Beckman Coulter Genomics, Danvers, MA, USA). The amplicon library was prepared using a TruSeq DNA sample preparation kit (Illumina Inc, San Diego, CA, USA). The libraries were sequenced using a 300PE MiSeq sequencing kit with the standard Illumina sequencing primers and the Illumina MiSeq platform (Illumina, CA, USA). Data processing was conducted as described previously (3, 17, 18). Briefly, paired-end reads were merged using FLASH v.1.2.11. The filtration of chimeric sequences and assignment of sequences with ≥ 97% similarity to operational taxonomic units (OTUs) were conducted using Vsearch software v.2.3.4. The representative sequence of each OTU was classified against the RDP database, Greengenes database and NCBI 16S Microbial database using QIIME’s parallel wrappers for the RDP classifier v.2.10.1. Beta diversity was analysed using QIIME v.1.9.1 and permutational analysis of variance (PERMANOVA).

### Quantitative PCR (qPCR) Primers and Cycling Conditions

The bacterial 16S rDNA gene copies in each sample were determined by qPCR using the 341f (5’-CCTACGGGNGGCWGCAG-3’) and 805r (5’- GACTACHVGGGTATCTAATCC-3’) primers. qPCR was performed using the ViiA7 Real-time PCR system (Applied Biosystems, Foster City, CA, USA). The qPCR reaction mixtures contained 5 μL of TB green, 0.4 μL (1 μM) of each primer, 0.2 μL of ROX reference dye, 2 μL of DNA template, and 2 μL of PCR-grade water. The reaction conditions comprised 30 s at 95°C, followed by 40 cycles of 5 s at 95°C and 34 s at 56°C, followed by 15 s at 95°C, 1 min at 60°C and 15 s at 95°C. qPCR calibration curves were constructed using linearized plasmid standards harbouring inserts of the PCR-amplified 16S rRNA gene of *Escherichia coli* ATCC 25922 as previously described (19, 20). Sample DNA was analysed in duplicate. The 16S rDNA copies in the saliva obtained by qPCR were converted to bacterial numbers based on the ratio of the 16S rDNA copies of ATCC 25922 obtained by qPCR to the numbers of ATCC 25922 copies obtained by culture using the same amount of bacterial cells as described previously (19).

### Metabolomics Profiling

For each 200 μL of salivary supernatant, 800 μL of methanol was added; the solution was thoroughly mixed before centrifugation at 14,000 rpm for 15 min, and the supernatant was filtered through a 0.22-μm membrane and treated as described previously (21, 22). After 20 μL of heptadecanoic acid (1 mg/mL) was added as an internal reference, the filtered supernatant was dried under nitrogen at room temperature, and 50 μL of methoxypyridine solution (15 mg/mL) was added. The solution was mixed for 1 min, sealed in an airtight container and kept at 37°C for 24 h. Next, 50 μL of N,O-bis(trimethylsilyl)acetamide with 1% trimethylchlorosilane was added, thoroughly mixed and kept for 2 h at 70°C for derivatization. The pretreated sample was analysed using an Agilent 7890A-5975C gas chromatography-mass spectrometry (GC-MS) system (Agilent, USA). The raw data obtained from GC-MS runs were analysed using Agilent Qualitative Analysis version B.07.00 software. The metabolites were identified using the NIST 17 database with a matching score of at least 80. The resulting dataset was exported and analysed using Umetrics SIMCA version 14.1 software.

### Cell Culture

OKF6/TERT2 (OKF6) keratinocytes from the Chinese Academy of Sciences (Shanghai, China) were grown in keratinocyte medium (ScienCell, Carlsbad, CA, USA) containing penicillin (100 U/mL) and streptomycin (100 μg/mL) at 37°C in a humidified 5% (v/v) CO_2_ incubator. The human monocyte cell line THP-1 was maintained at 0.3–0.8×10^6^ cells/mL in RPMI 1640 supplemented with 10% foetal bovine serum (FBS), HEPES (25 mM), 2-mercaptoethanol (0.05 mM), penicillin (100 U/mL) and streptomycin (100 μg/mL).

### Coculture of Salivary Microbes or Supernatants With Cells

OKF6 or THP-1 cells were cultured overnight at 4×10^4^ cells per well in a 96-well plate. THP-1 cells were differentiated into macrophages (dTHP-1) by treatment with 100 ng/mL of phorbol 12-myristate 13-acetate (Sigma-Aldrich) and 0.3% bovine serum albumin in serum-free RPMI 1640 for 48 h; next, dTHP-1 cells were further cultured overnight in RPMI 1640 supplemented with 10% FBS, penicillin (100 U/mL), and streptomycin (100 μg/mL). To inactivate saliva microbes, the pellets from saliva were washed, resuspended in PBS (pH=7.4) and incubated in an 80°C water bath for 15 min. After determining the concentrations using a Thoma cell-counting chamber, salivary microbes were added to OKF6 cell cultures at a multiplicity of infection of 50. Additionally, salivary supernatants sterilized by filtration were separately added to other OKF6 or dTHP-1 cultures. The supernatants of these cocultures were sampled at 0 and 48 h for cytokine assays.

### Ultrasonic Scaling

Calculus and plaque that attached to tooth surfaces were removed by ultrasonic crushing using a periodontal ultrasonic scaler (Satelec, Mérignac, France), while those embedded deeply were removed by curettage.

### Statistical Analysis

Regarding the results of liver functions, cytokines, metabolites and alpha diversity of the microbiota, the Mann-Whitney U test was used to compare any two datasets that were not normally distributed; otherwise, one-way ANOVA followed by the Student-Newman-Keuls method was used. The Wilcoxon rank-sum test combined with the Benjamini-Hochberg method was applied to compare bacterial taxa. The Spearman’s rank correlation test was used to analyse the correlations. For all graphs, nonsignificant results (*P* > 0.05) were left blank; *P < 0.05; **P < 0.01 and ***P < 0.001; #*P*_adj_ < 0.05; ##*P*_adj_ < 0.01 and ###*P*_adj_ < 0.001.

## Results

### Characteristics of PBC Patients and Healthy Controls

Of 602 outpatients diagnosed with PBC, 528 were excluded because they also had non-PBC diseases, among which the top ten were autoimmune hepatitis, chronic gastritis, chronic hepatitis B, fatty liver disease, Sjogren’s syndrome, hypertension, diabetes, connective tissue disease, thyroid disease, and chronic kidney disease, This, 74 patients met the inclusion criteria. Finally, 39 patients agreed to participate in the present study, and 37 age- and sex-matched healthy controls (HCs) were enrolled. Compared with HCs, the levels of blood total protein, globulin, alanine aminotransferase (ALT), aspartate aminotransferase (AST), alkaline phosphatase (ALP), total bilirubin, direct bilirubin, indirect bilirubin, γ-glutamyl transpeptidase (GGT) and eosinophils were increased in PBC patients (**Table 1**). The median Mayo risk score was 3.41 for PBC patients.

**Table 1:** Clinical features of PBC patients and HCs in this study.

	PBC (n=35)	HC (n=37)	(*P* values)
Age (years)	50.91 ± 1.42	52.59 ± 1.19	0.24
Males/females	2/33	2/35	–
Mayo risk score	3.41 (3.06, 3.79)	–	–
Total protein (g/L)	80.48 ± 1.01	74.43 ± 0.67	1.20E-5
Globulin (g/L)	34.53 ± 1.24	26.91 ± 0.55	1.34E-6
ALT (U/L)	33.44 ± 3.07	17.59 ± 1.59	3.68E-5
AST (U/L)	44.56 ± 5.11	19.5 ± 0.99	3.12E-05
ALP (U/L)	157.97 ± 16.11	74.86 ± 5.25	1.85E-5
Total bilirubin (mg/dL)	0.85 (0.61, 1.17)	0.58 (0.41, 0.73)	1.00E-03
Direct bilirubin (mg/dL)	0.29 (0.18, 0.39)	0.18 (0.12, 0.29)	3.90E-02
Indirect bilirubin (mg/dL)	0.68 ± 0.07	0.38 ± 0.01	4.45E-04
GGT (U/L)	154.72 ± 31.85	18.77 ± 1.98	1.74E-04
Eosinophil (%)	2.55 ± 0.44	1.38 ± 0.15	1.70E-02
Eosinophil (10^9^/L)	0.13 ± 0.02	0.07 ± 0.01	1.59E-02

ALT, alanine aminotransferase; AST, aspartate aminotransferase; ALP, alkaline phosphatase; GGT, γ-glutamyl transpeptidase.

### The Salivary Microbiota Is Significantly Different Between PBC and HCs

**Figure 1:**
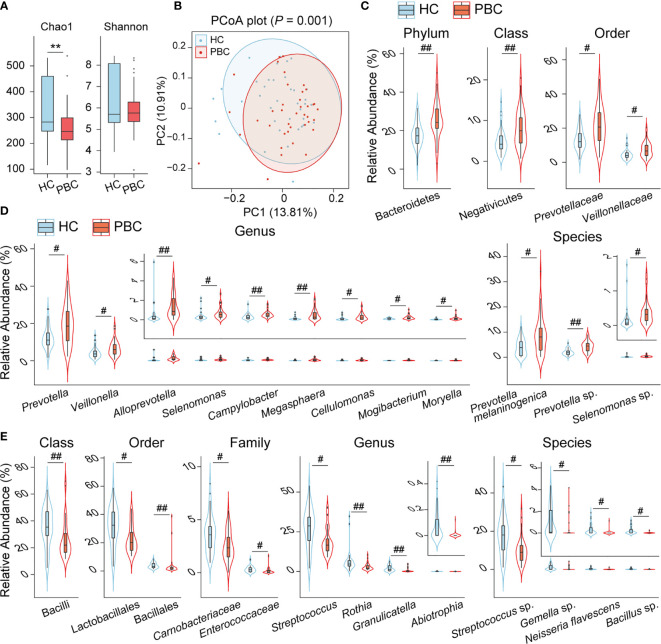
The salivary microbiota of PBC patients is distinct from that of HC subjects. **(A)** Box plot and distribution of the Chao1 index and Shannon index. **(B)** PCoA plot based on the unweighted UniFrac distance. **(C)** Phyla, classes, and orders enriched in PBC saliva. **(D)** Genera and species enriched in PBC saliva. **(E)** Classes, orders, families, genera and species depleted in PBC saliva. **P < 0.01; ^#^ *P*_adj_ < 0.05; ^##^ *P*_adj_ < 0.01.

To explore the differences between the salivary microbiota of PBC patients and HCs, we conducted 16S metagenomic sequencing and obtained 2,427,150 reads from 39 PBC salivary samples and 2,494,209 reads from 37 HC salivary samples. Bacteria from 17 phyla and 837 species, accounting for 10^-1^ to 10^-7^ of the total sequences, were identified. The bacterial community richness, as estimated by the Chao1 index, was lower in PBC saliva than in HC saliva, while the salivary bacterial community diversity, as characterized by the Shannon index, showed no significant differences between the two groups (**Figure 1A**). Principal coordinate analysis (PCoA) demonstrated separation in the salivary microbiota composition between PBC and HC individuals (**Figure 1B**), which was confirmed by permutational multivariate analysis of variance (PERMANOVA) (*P* = 0.001).

Comparing the salivary bacterial taxa of PBC patients with those of HCs, three main features of their differences were observed. First, the phylum Bacteroidetes was enriched in PBC saliva versus HC saliva. This difference was mainly due to the enrichment of *Prevotellaceae* at the family level, *Prevotella* at the genus level, and both *Prevotella melaninogenica* and *Prevotella* sp. at the species level, although the genus *Alloprevotella* was also enriched as well (**Figures 1C, D**). Second, the phylum Firmicutes was depleted (*P* = 0.014, *P*_adj_ = 0.12) in PBC saliva due to the substantial depletion of certain taxa of the class Bacilli and the slight enrichment of members of the classes Clostridia and Negativicutes. The order Lactobacillales, the genus *Streptococcus* and the unclassified species *Streptococcus* sp. contributed substantially to the depletion of the class Bacilli. Additionally, the order Bacillales, family *Carnobacteriaceae* and *Enterococcaceae*, genus *Abiotrophia* and *Granulicatella*, and species *Bacillus* sp. belonging to Bacilli were also depleted in PBC saliva. Among the PBC-enriched salivary members of the class Negativicutes, *Veillonellaceae and Veillonella* were the most enriched families and genera. However, none of the 18 species identified in our study belonging to *Veillonella* were significantly different between the saliva of PBCs and HCs, indicating that *Veillonella* enrichment was a joint contribution of its species. Furthermore, several taxa with relative abundances lower than 1% and belonging to the class Negativicutes, such as the genera *Megasphaera*, *Mogactrium*, *Moryela*, and *Selenomonas*, were enriched in PBC saliva. Additionally, the genera *Mogibacterium* and *Moryella* belonging to the class Clostridia were also enriched (**Figure 1D**). Third, several taxa of the phylum Actinobacteria or Proteobacteria were also altered. The former includes the depletion of *Rothia* and enrichment of *Cellulomonas*, and the latter includes the depletion of *Neisseria flavescens* and enrichment of *Campylobacter* in PBC saliva (**Figures 1D, E**).

### sCD163 Is Enriched, While MIF, IL-16 and CCL25 Are Depleted, in PBC Saliva

**Figure 2:**
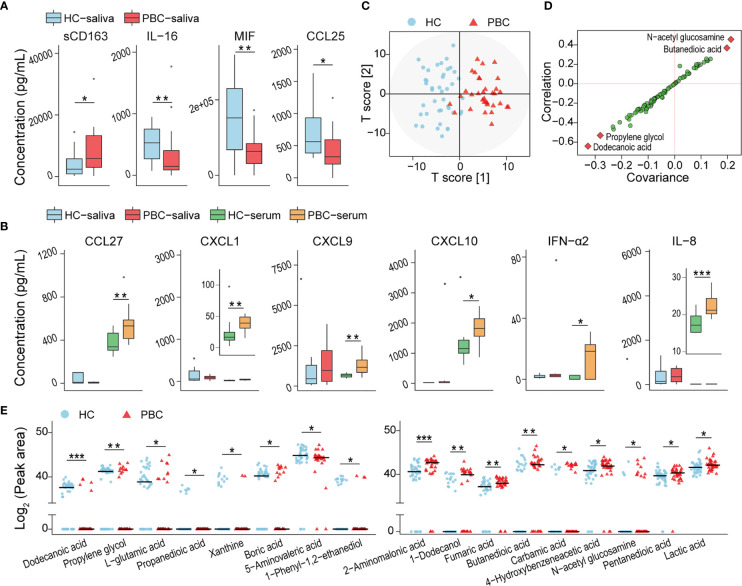
Inflammatory cytokines and harmful metabolites were enriched in PBC saliva compared with that in HC saliva. **(A)** Inflammatory cytokines were distributed differently in PBC saliva and HC saliva. **(B)** The levels of certain cytokines with nonsignificant differences between HC and PBC saliva were increased significantly in PBC serum. **(C)** OPLS-DA plots of the metabolome illustrating that PBC and HC subjects were clearly separated. **(D)** S-plot highlighting four salivary metabolites as potential biomarkers. **(E)** Seventeen salivary metabolites were different in peak areas and/or detection rates between HC and PBC samples. *P < 0.05.

The concentration of 80 salivary soluble cytokines was tested to assess the changes in the oral immunity of PBC patients that may be related to the altered salivary microbiota. The concentration of the soluble cluster of differentiation 163 (sCD163) was higher, while that of macrophage MIF, IL-16, and thymus-expressed chemokine (TECK)/CCL25 was lower in PBCs than in HCs (**Figure 2A**). We also found that some cytokines, such as IL-8, chemokine (C-C motif) ligand 27 (CCL27), C-X-C motif chemokine 1 (CXCL1), CXCL9, CXCL10 and IFN-α2, which were not significantly different in HC and PBC saliva, were significantly enriched in PBC serum (**Figure 2B**). This finding indicated that the alterations in salivary cytokines most likely originated from oral sources but not from bleeding.

### Salivary Metabolome Dysbiosis in PBC Patients

Eighty-six compounds were identified from the saliva of patients with PBC and HCs by GC-MS. The orthogonal projection to latent structures with discriminant analysis (OPLS-DA) illustrated that the metabolic profiles of PBC and HC saliva were clearly separated (**Figure 2C**). The variable influence on projection (VIP) of ten metabolites (dodecanoic acid, propylene glycol, L-glutamic acid, 3-hydroxybutyric acid, N-acetyl glucosamine, pyrimidine, butanedioic acid, 2-hydroxyisocaproic acid, 1-phenyl-1,2-ethanediol, boric acid) was higher than 1.5, indicating that these metabolites contribute substantially to the OPLS-DA model in separating PBC and HC salivary metabolomes. The S-plot indicated N-acetyl glucosamine, butanedioic acid, propylene glycol, and dodecanoic acid as potential biomarkers (**Figure 2D**).

Next, we compared the abundances of each identified compound in the saliva of PBC patients and HCs. Compared with those in HC saliva, nine salivary metabolites, including three carboxylic acids (pentanedioic acid, butanedioic acid, fumaric acid), two substituted carboxylic acids (lactic acid, 4-hydroxybenzeneacetic acid), 2-aminomalonic acid, 1-dodecanol, carbamic acid, and N-acetyl-glucosamine, were enriched in PBC saliva. Additionally, eight metabolites, including two carboxylic acids (dodecanoic acid and propanedioic acid), two amino acids (L-glutamic acid and 5-aminovaleric acid), two alcohols (propylene glycol and 1-phenyl-1,2-ethanediol), xanthine, and boric acid, were depleted in PBC saliva (**Figure 2E**).

### sCD163, 4-Hydroxybenzeneacetic Acid and 2-Aminomalonic Acid Are Significantly Correlated With Salivary Cytokines, Bacteria and Metabolites

To explore the correlations among PBC-altered bacteria, metabolites and cytokines in saliva, we conducted Spearman’s correlation analysis. The absolute value of the correlation coefficient r > 0.4 and *P* < 0.01 were used as a screening threshold to show the extremely significant results (23).

**Figure 3:**
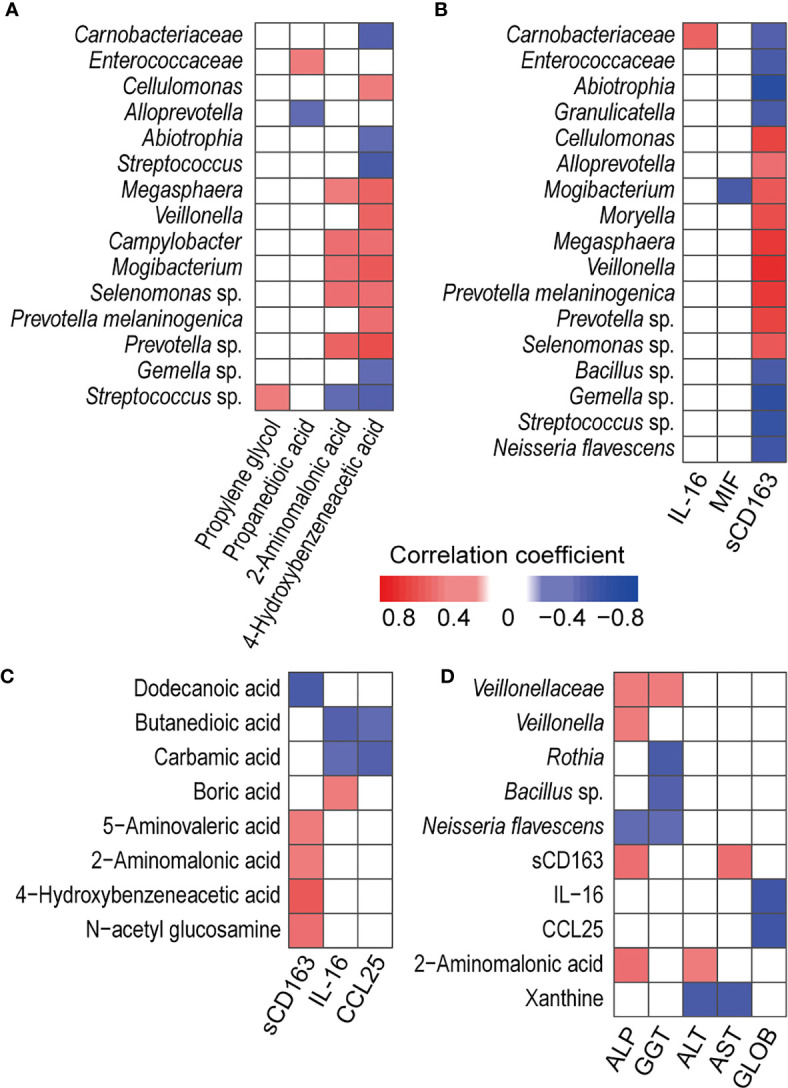
Associations between PBC-altered salivary cytokines, metabolites, and bacteria and their associations with haematological variables or liver functions. **(A)** Association of PBC-altered salivary bacteria with metabolites. **(B)** Association of PBC-altered salivary bacteria with cytokines. **(C)** Association of PBC-altered salivary metabolites with cytokines. **(D)** Association of PBC-altered salivary bacteria, cytokines and metabolites with haematological variables or liver functions. Both correlation coefficients (in absolute value) higher than 0.4 and *P* < 0.01 were used as significance thresholds.

Correlation analysis of salivary bacteria with the metabolites revealed that PBC-enriched salivary 4-hydroxybenzeneacetic acid and 2-aminomalonic acid were positively correlated with PBC-enriched salivary *Campylobacter*, *Mogibacterium*, *Megasphaera*, *Prevotella* sp., and *Selenomonas* sp. (**Figure 3A**). 4-Hydroxybenzeneacetic acid was also positively correlated with PBC-enriched *Cellulomonas*, *Veillonella*, and *Prevotella melaninogenica* but was negatively correlated with PBC-depleted *Carnobacteriaceae*, *Abiotrophia*, *Streptococcus*, *Gemella* sp., and *Streptococcus* sp. Furthermore, *Streptococcus* sp. was negatively correlated with 2-aminomalonic acid and positively correlated with propylene glycol. Propanedioic acid was negatively correlated with *Alloprevotella* and positively correlated with *Enterococcaceae*.

PBC-altered salivary bacteria and metabolites were mainly correlated with PBC-enriched salivary sCD163. First, sCD163 was positively correlated with *Alloprevotella*, *Cellulomonas*, *Megasphaera*, *Mogibacterium*, *Moryella*, *Veillonella*, *Prevotella melaninogenica*, *Prevotella* sp., and *Selenomonas* sp. but negatively correlated with *Enterococcaceae*, *Abiotrophia*, *Carnobacteriaceae*, *Granulicatella*, *Bacillus* sp., *Gemella* sp., *Neisseria flavescens* and *Streptococcus* sp. The correlation coefficients of sCD163 with *Megasphaera*, *Veillonella*, and *Prevotella melaninogenica* were higher than 0.65, indicating high correlations (**Figure 3B**). Second, salivary sCD163 was positively correlated with PBC-enriched salivary metabolites 4-hydroxybenzeneacetic acid, 2-aminomalonic acid, N-acetyl glucosamine, and 5-aminovaleric acid and negatively correlated with PBC-depleted dodecanoic acid. Additionally, *Selenomonas* sp. was positively correlated with the Mayo risk score, while butanedioic acid was negatively correlated with IL-16 and CCL25 (**Figure 3C**).

### Salivary Veillonellaceae Members, 2-Aminomalonic Acid, and sCD163 Are Positively Correlated With Liver Function Indicators

Under the significance threshold of both correlation coefficients (in absolute value) higher than 0.4 and *P* < 0.01, PBC-enriched Negativicutes and its members *Veillonellaceae* and *Veillonella* were positively correlated with serum ALP and GGT (**Figure 3D**). *Rothia* and *Bacillus* sp. sp. were negatively correlated with GGT. Additionally, PBC-enriched salivary sCD163 was positively correlated with serum ALP and AST, while salivary PBC-depleted salivary IL-16 and CCL25 were negatively correlated with serum globin. 2-Aminomalonic acid was positively correlated with ALP and ALT, while salivary xanthine was negatively correlated with serum ALT and AST.

### PBC Salivary Microbes Induce More sIL-6Rα, sIL-6Rβ and TNFSF13B From OKF6 Keratinocytes

**Figure 4:**
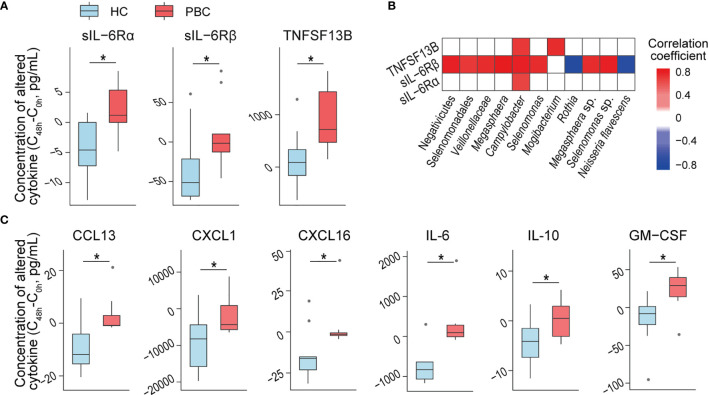
PBC salivary microbes and supernatants induce more inflammatory cytokine secretion than HC salivary microbes from keratinocytes and THP-1 macrophages. **(A)** Inflammatory cytokine concentrations that were differentially expressed in OKF6 cells after coculture with HC or PBC salivary microbes. **(B)** Association of the relative abundance of salivary microbes with altered cytokine concentrations after the coculture of OKF6 with HC or PBC salivary microbes. **(C)** Inflammatory cytokine concentrations that were differentially expressed in THP-1 macrophages after coculture with HC or PBC salivary supernatants. *P < 0.05.

Salivary microbes may directly interact with epidermal cells. Therefore, we evaluated their effects on OKF6 keratinocytes, which represent the major members of oral epithelial cells and are a model system to analyse host–microbe interactions. To prevent alterations in the microbiota composition during coculture with OKF6 cells, we thermoinactivated the purified salivary microbes. After 48 h of coculturing heat-inactivated salivary microbes with OKF6, we detected higher increases in sIL-6Rα, sIL-6Rβ, and TNFSF13B in the culture media with added PBC salivary microbes than in those with added HC microbes (**Figure 4A**).

To explore the potential roles of salivary bacteria in immune regulation, we correlated PBC-altered bacteria with the above cytokines. At the *P* < 0.01 level, sIL-6Rα, sIL-6Rβ and TNFSF13B were correlated with *Campylobacter*. Furthermore, sIL-6Rβ was positively correlated with *Veillonellaceae*, *Selenomonas* sp., and *Megasphaera* sp. but negatively correlated with *Rothia* and *Neisseria flavescens*. Additionally, TNFSF13B was positively correlated with *Mogibacterium*. The absolute values of all the correlation coefficients were higher than 0.6, indicating a high correlation (**Figure 4B**).

When cocultured with OKF6, only a few bacteria-free salivary supernatants induced inflammatory cytokines, which showed no significant differences in distribution between HC and PBC samples, indicating good tolerance of oral keratinocytes to salivary supernatants.

### PBC Salivary Supernatant Induces More Cytokines From THP-1 Macrophages

Some molecules in the salivary supernatant can penetrate into the oral mucosal lamina propria, and macrophages provide a first line of defence if this breach occurs. When cocultured with THP-1 macrophages, bacteria-free PBC salivary supernatants induced higher increases in the concentrations of six cytokines than HC salivary supernatants. Three were chemokines (CCL13, CXCL1 and CXCL16) that can induce one or more leukocytes to participate in inflammation. The other three cytokines are the well-known proinflammatory cytokine IL-6, anti-inflammatory cytokine IL-10 and GM-CSF (**Figure 4C**). These results indicate that PBC salivary supernatants have stronger proinflammatory capacity than HC salivary supernatants and may be involved in disease progression. However, none of the alterations in the above cytokines were significantly correlated with the concentration of PBC-altered salivary cytokines and metabolites, indicating which substances in PBC salivary supernatant induce more cytokines warrant further study.

### Toothbrushing Breaks the Microbiota and Depletes More Inflammatory Cytokines and Harmful Metabolites From PBC Saliva

**Figure 5:**
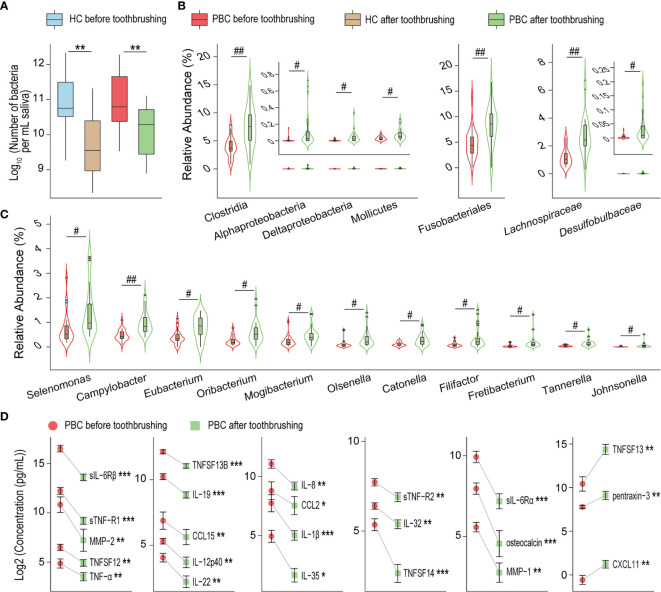
Toothbrushing reduces the number of inflammatory cytokines in PBC saliva more than that in HC saliva. **(A)** Number of bacteria per millilitre of saliva in PBC patients and healthy controls before and after toothbrushing. **(B)** and **(C)** Bacterial taxa with higher relative abundances in PBC saliva after toothbrushing than before toothbrushing. **(D)** Inflammatory cytokines that were significantly reduced from before to after toothbrushing in PBC saliva. *P < 0.05; **P < 0.01; ***P < 0.001; ^#^ *P*_adj_ < 0.05; ^##^ *P*_adj_ < 0.01.

Toothbrushing reduced the salivary bacterial burden by nearly 90% in both PBC patients and HCs (**Figure 5A**). Additionally, the composition of salivary microbiota was significantly different before versus after toothbrushing in PBC patients but not in HCs. Most altered salivary taxa after toothbrushing in PBC showed an increase in their relative abundance (**Figures 5B, C**), including the order Fusobacteriales, the classes Clostridia, Mollicutes, Alphaproteobacteria and Deltaproteobacteria, the families *Lachnospiraceae* and *Desulfobulbaceae*, and the genera *Campylobacter*, *Catonella*, *Eubacterium*, *Filifactor*, *Fretibacterium*, *Johnsonella*, *Mogibacterium*, *Olsenella*, *Oribacterium*, *Selenomonas*, and *Tannerella*. However, in general, the amount of each bacterial taxon mentioned above was also sharply reduced after toothbrushing.

To explore the effect of salivary microbiota on oral immunity, we compared the concentrations of inflammatory cytokines before and after toothbrushing using paired-sample analysis (**Figure 5D**). Twenty cytokines were decreased in PBC saliva, including TNF-related cytokines (sTNF-R1, sTNF-R2, TNF-α, TNFSF12, TNFSF13B, TNFSF14), chemokines (CCL15, CCL2, IL-8/CXCL8), IL-12 family cytokines (IL-12p40, IL-35), IL-20 family cytokines (IL-19, IL-22), metalloproteases (MMP-1, MMP-2), soluble IL-6 receptors (sIL-6Rα and sIL-6Rβ), IL-1β, IL-32, and osteocalcin. Conversely, three cytokines were increased in PBC saliva: CXCL11, pentraxin-3 and TNFSF13. However, none of the concentrations of the tested salivary cytokines were altered in HCs before and after toothbrushing.

**Figure 6:**
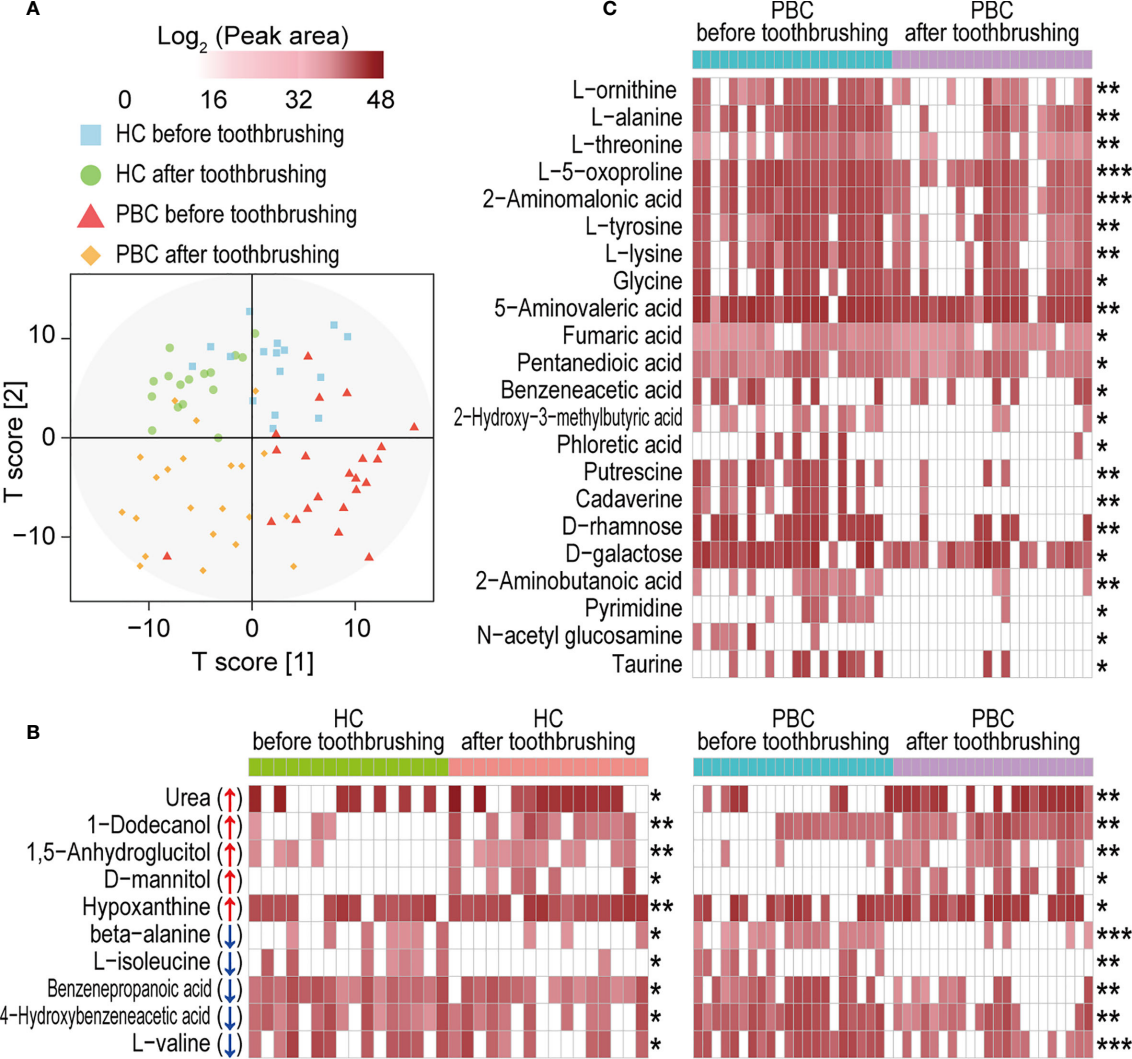
Toothbrushing reduces the number of metabolites in PBC saliva more than that in HC saliva. **(A)** OPLS-DA plot of the salivary metabolome profile in PBC and HC saliva before and after toothbrushing. **(B)** Metabolites that were significantly altered in both PBC and HC saliva from before to after toothbrushing. **(C)** Metabolites that were significantly altered only in PBC saliva from before to after toothbrushing. *P < 0.05; **P < 0.01 and ***P < 0.001.

PBC salivary metabolome profiles before and after toothbrushing clustered more separately than those of HCs (**Figure 6A**). Toothbrushing caused alterations in the abundances of a total of 32 metabolites in the saliva of PBC patients and/or HCs. Ten metabolites changed similarly in both PBC and HC saliva from before to after toothbrushing (**Figure 6B**). Among them, 1-dodecanol, urea, 1,5-anhydroglucitol, D-mannitol, and hypoxanthine increased, while L-valine, beta-alanine, 4-hydroxybenzeneacetic acid, benzenepropanoic acid and L-isoleucine decreased after toothbrushing. However, 22 metabolites decreased only in PBC saliva after toothbrushing (**Figure 6C**). These metabolites include ten amino acids (2-aminomalonic acid, 2-aminobutanoic acid, 5-aminovaleric acid, L-alanine, glycine, L-lysine, L-tyrosine, L-threonine, L-ornithine, and L-5-oxoproline), five carboxylic acids (fumaric acid, pentanedioic acid, benzeneacetic acid, and 2-hydroxy-3-methylbutyric acid, and phloretic acid), two amines (cadaverine and putrescine), two monosaccharides (D-rhamnose and D-galactose), and pyrimidine, N-acetyl glucosamine and taurine. Among them, the mean abundance of cadaverine, phloretic acid, putrescine, and pyrimidine decreased by more than 90% from before to after toothbrushing.

### ALP and Bilirubin in PBC Serum Decrease After Full-Mouth Scaling and Root Planing

Two months after full-mouth scaling and root planing of the patient volunteers, ALP decreased in 85.33% of the patients; total bilirubin and indirect bilirubin decreased in 71.42% but remained unchanged in 14.29% of the patients; direct bilirubin decreased in 57.14% but remained unchanged in 28.57% of the patients (**Table 2**). These results suggested that alterations in the oral microbiota are one reason for liver damage in PBC patients.

**Table 2:** Decreased levels of ALP and bilirubin in PBC serum after full-mouth scaling and root planing.

	Mean ± standard error of mean (n=7)		Decreased patients (%)	Unchanged patients (%)	Increased patients (%)
	Before SRT	After SRT			
ALP (U/L)	126.43 ± 22.74	109.86 ± 21.09	85.71	0	14.29
TB (mg/dL)	0.65 ± 0.08	0.57 ± 0.05	71.42	14.29	14.29
DB (mg/dL)	0.29 ± 0.05	0.25 ± 0.0.03	57.14	28.57	14.29
IB (mg/dL)	0.36 ± 0.04	0.32 ± 0.03	71.42	14.29	14.29

SRT, full-mouth scaling and root planing; ALP, alkaline phosphatase; TB, total bilirubin; DB, direct bilirubin; IB, indirect bilirubin.

## Discussion

The role of the human microbiota in autoimmune diseases has attracted increased attention (24). However, how the oral microbiota affects PBC is not fully understood. The present study demonstrated not only alterations in the PBC salivary microbiota characterized by enrichment of bacteria such as the phylum Bacteroidetes and genus *Campylobacter* and depletion of bacteria such as the genera *Streptococcus* and *Rothia* but also the enrichment of metabolites such as 2-aminomalonic acid and the upregulation of inflammatory cytokines such as sCD163 in PBC saliva. The altered salivary microbiota, cytokines, and metabolites were associated with not only each other but also liver function parameters. After toothbrushing, 20 cytokines and 18 metabolites were depleted in PBC saliva, while none of the detected cytokines or metabolites were altered in HC saliva. PBC salivary microbes induced more sIL-6Rα, sIL-6Rβ, and TNFSF13B from oral keratinocyte OKF6 cells than HC salivary microbes, but PBC salivary supernatants induced more IL-6, IL-10, GM-CSF, CCL13, CXCL1 and CXCL16 from THP-1 macrophages than those of HCs. Importantly, full-mouth scaling and root planing improved the liver functions of PBC patients.

Alterations in the PBC salivary microbiota have several distinctive features, although comparisons among different studies may limit the results. First, some alterations of the PBC salivary microbiota differ from those of several known liver diseases. For example, compared with HC saliva, PBC saliva showed depleted *Enterococcaceae* and *Streptococcaceae* and enriched *Veillonellaceae*, but the saliva of hepatitis C virus/alcohol/nonalcoholic steatohepatitis cirrhosis subjects showed enriched *Enterococcaceae*, with no alterations in *Streptococcaceae* and *Veillonellaceae* (25). *Porphyromonas gingivalis*, a keystone periodontal pathogen that can induce nonalcoholic fatty liver disease in murine models, was not enriched in PBC saliva (26). Second, some alterations of the PBC salivary microbiota differ from those of several autoimmune diseases. For example, compared with HC saliva, saliva of celiac disease patients (27) and rheumatoid arthritis patients (28, 29) showed enriched *Lactobacillus* and *Lactobacillus salivarius* separately, which were not observed in the comparison between PBC patients and HCs; patients with rheumatic heart disease had enriched salivary *Streptococcus* and depleted salivary *Prevotella* and *Veillonella*, which were altered conversely in PBC patients (30); Bacteroidetes were enriched in PBC saliva but were depleted in the saliva of patients with autoimmune polyendocrine syndrome type-1 (31). Finally, compared with HCs, PBC patients exhibited depletion of salivary Lactobacillales, particularly *Streptococcaceae*, which was enriched in PBC faeces (3, 4).

Our results indicate that PBC saliva has some alterations that potentially increase their pathogenicity. First, in PBC saliva, some enriched bacterial taxa, such as *Prevotellaceae*, *Veillonella* and *Campylobacter*, are rich in opportunistic pathogens that may have already been involved in PBC development or may have continuously impaired the health of PBC patients. For example, microabscesses were occasionally observed in the livers of animals infected by *Campylobacter concisus* (32). *Prevotella melaninogenica* is commonly cultured as the sole infectious agent in extraoral abscesses such as spondylitis, osteomyelitis, pyomyositis, peritoneal abscesses and vaginal mesh infections (33). *Veillonella* members are frequently associated with mixed infections and are recovered mixed with mouth and bowel flora (34). Consistent with this report, we found that *Veillonellaceae* and *Veillonella* were positively correlated with serum ALP and GGT, adding to evidence of harmful effects of these bacteria on PBC. Second, some PBC-altered salivary cytokines may affect the health of the oral tract and even the whole digestive tract. For example, sCD163 enriched in PBC saliva is a marker of M2 macrophage activation and is involved in the pathogenesis of autoimmune diseases, atherosclerosis, diabetes and cancer (35–37); in PBC patients, serum sCD163 was reported as a noninvasive marker of liver disease severity and prognosis (36, 38). Correspondingly, our results showed that PBC-enriched salivary sCD163 was not only intensively linked with salivary bacteria and metabolites but also positively correlated with serum ALP and AST. We also found that bacteria-free PBC salivary supernatants induced THP-1 macrophages to produce more sCD163 (*P* = 0.09) than HC salivary supernatants in the coculture experiment. This insignificant result may be due to the cell model type and concentration of active molecules in the coculture. Third, some PBC-enriched salivary metabolites are involved in health damage. For example, pentanedioic acid induces the death of neuronal cells and results in glutamic acidaemia I under pathological conditions (39). 2-Aminomalonic acid is associated with genotoxicity; furthermore, aminomalonate building block was uniquely incorporated into the unprecedented macrocyclic structure of the genotoxic precolibactin-886 biosynthesized by *Escherichia coli* (40). Taken together, these PBC-enriched bacteria, proinflammatory cytokines and bacterial products may contribute to the development of PBC, a finding that requires further exploration.

Our results partially verified the PBC salivary microbiota as an important cause of oral inflammation and PBC development. First, reducing salivary microbes caused a significant decrease in salivary cytokines in PBC. After toothbrushing, we found that the salivary bacterial burden decreased nearly by 90% in both PBC patients and HCs. Additionally, twenty inflammatory cytokines, such as TNF-α, IL-8 and IL-1β, and eighteen salivary harmful metabolites, including cadaverine and putrescine, were reduced in PBC saliva but not in HC saliva during this process. Because the interactions between the multilayer epithelium and microbiota shape oral homeostasis (41), these results indicated that salivary microbiota and their products contribute to the occurrence and maintenance of oral inflammation in PBC patients. Second, *in vitro*, salivary microbes and their products from PBC patients induced more cytokines from cocultured cells. Our results showed that bacteria-free PBC salivary supernatants induced higher increases in the concentrations of IL-6, IL-10, GM-CSF, CCL13, CXCL1 and CXCL16 from THP-1 macrophages than HC salivary supernatants; additionally, PBC salivary microbes induced more sIL-6Rα, sIL-6Rβ and TNFSF13B from OKF6 keratinocytes than HC salivary microbes. Correspondingly, in a previous study, lipopolysaccharide of *Veillonella parvula*, a member of PBC-enriched *Veillonella*, stimulated TNF-α and IL-6 release in human peripheral blood mononuclear cells in a dose-dependent manner (42). Therefore, these *in vitro* experiments may partially prove that the microbiota of PBC patients and its products have a stronger pathogenic effect than those of HCs. Third, full-mouth scaling and root planing, which can reduce oral adherent microbes and their products, reduce the ALP and bilirubin levels in PBC serum. The levels of ALP and bilirubin can predict the outcomes (liver transplantation or death) of patients with PBC and may be used as surrogate end points in therapy trials (43). Therefore, this finding indicated that full-mouth scaling and root planing may contribute to the treatment of PBC.

Our results suggest that several pathways may be involved in the interaction between salivary microbiota and PBC patients. First, the IL-6 pathway may be involved in this process. IL−6 can signal *via* the membrane-bound and soluble IL−6 receptor (IL−6R); classic signalling *via* the membrane-bound receptor primarily located on the surface of hepatocytes and leukocytes is mainly responsible for the regenerative and antibacterial effects of IL-6, whereas trans-signalling *via* the soluble receptor mainly accounts for the inflammation of cells that did not express IL−6R, such as endothelial cells and smooth muscle cells (44). Our results showed that sIL-6Rα and sIL-6Rβ were decreased in PBC saliva after toothbrushing, PBC salivary microbes induced more sIL-6Rα and sIL-6Rβ from OKF6 keratinocytes than from HCs, and PBC salivary supernatant induced more IL-6 from THP-1 macrophages. These findings indicate the involvement of both classic signalling and trans-signalling of IL-6. Correspondingly, inhibition of IL-6 trans-signalling is sufficient to block inflammatory bowel disease and rheumatoid arthritis in mouse models (45). Second, TNF signalling may play a role in the salivary microbe-host interaction in PBC patients. We found that PBC salivary microbes induced more TNFSF13B from OKF6 keratinocytes than HCs; additionally, toothbrushing reduced TNF-α, TNFSF12, TNFSF13B and TNFSF14 in only in PBC saliva, indicating the potential participation of TNF superfamily signalling (46). Several TNF superfamily molecules mediate autoimmunity. For example, TNFSF13B induces symptoms resembling those of human Sjogren’s syndrome and SLE when overexpressed in mice (47). Additionally, we found that toothbrushes reduced salivary sTNF-R1 and sTNF-R2 in PBC patients, suggesting the involvement of reverse TNF signalling, where sTNFR1 and sTNFR2 bind to plasma membrane-bound TNF-α to activate reverse (outside-to-inside) TNF signalling in immune cells (48). Third, toothbrushing reduced salivary CXCL8 in PBC patients and PBC salivary supernatants induced more CXCL1 (belonging to the CXCL8 family) in THP-1 macrophages than in HCs. These two results suggest a potential contribution of the CXCL1/8-CXCR1/2 axis to the interaction between salivary microbiota and PBC patients (49). Although the abovementioned pathways obtained from our different level results are inferentially limited, they provide some potential ideas for future research.

Our results suggest that maintaining oral microbiota health is important for the treatment and even prevention of PBC. Increasing levels of oral inflammation, harmful microbes and metabolites can aggravate PBC and may form a vicious cycle with deteriorating liver functions, which must be monitored and intervened without delay. Importantly, saliva, particularly that sampled before toothbrushing after waking up, is a simple and appropriate indicator for oral and even general health and warrants further research and development. Classical oral care, such as brushing, flossing and dental visits, has been recognized as indispensable way to maintain health and prevent disease in not only the oral cavity but also the whole body (11). Our results demonstrate that daily toothbrushing and regular scaling can alleviate oral and systemic inflammation and partially improve liver function in PBC patients. In the future, treatment based on oral microbiota, such as oral probiotics and oral microbiota transplantation, will also be candidates to prevent and treat PBC.

In summary, we found significant alterations in the composition of salivary bacteria, metabolites and inflammatory cytokines in PBC patients. The PBC microbiota and their products showed more significant inflammation-inducing effects *in vitro* and *in vivo* than those of HCs. Full-mouth scaling and root planing partially improved the liver function of patients. This study provides useful insights into the pathogenesis and comprehensive treatment of PBC.

## Data Availability Statement

The datasets presented in this study can be found in online repositories. The names of the repository/repositories and accession number(s) can be found below: https://www.ncbi.nlm.nih.gov/bioproject/PRJNA732238, PRJNA732238.

## Ethics Statement

The ethics committee of Zhejiang University approved this study. The patients/participants provided their written informed consent to participate in this study.

## Author Contributions

Study concept and design: LXL, HJ, HD, and LJL. Acquisition and analysis of data: LXL, RY, HJ, XC, QW, KW, JY, YTL, DF, YFL, LY, SG, JC, and HD. Drafting of the manuscript: LXL, RY, HJ, XC, QW, and KW. Critical revision of the manuscript for important intellectual content: LXL, RY, HJ, HD, and LJL. Statistical analysis: LXL, RY, HJ, QW, and KW. All authors contributed to the article and approved the submitted version.

## Funding

This research is supported by the National Key Research and Development Program of China (2018YFC2000500), National Natural Science Foundation of China (81570512, 81330011), and Natural Science Foundation of Zhejiang Province, China (LQ19H030007).

## Conflict of Interest

The authors declare that the research was conducted in the absence of any commercial or financial relationships that could be construed as a potential conflict of interest.

## Abbreviations

PBC, primary biliary cholangitis; UDCA, ursodeoxycholic acid; PDC-E2, pyruvate dehydrogenase E2 components; HC(s), healthy control(s); ALT, alanine aminotransferase; AST, aspartate aminotransferase; ALP, alkaline phosphatase; GGT, γ-glutamyl transpeptidase; PDW, platelet distribution width; CCL, chemokine (C-C motif) ligand; CX3CL1, chemokine (C-X3-C motif) ligand 1; CXCL, C-X-C motif chemokine; GM-CSF, granulocyte-macrophage colony-stimulating factor; IFN, interferon; IL, interleukin; MMP-1, matrix metalloproteinase-1; sCD163, soluble cluster of differentiation 163; sIL-6Rα, soluble IL-6 receptor α; sIL-6Rβ, soluble IL-6 receptor β; sTNFR, soluble tumour necrosis factor receptor; TNFSF, tumour necrosis factor ligand superfamily member; OPLS-DA, the orthogonal projection to latent structures discriminant analysis; MIF, macrophage migration-inhibitory factor.
